# Biomarkers for Renal Cell Carcinoma Recurrence: State of the Art

**DOI:** 10.1007/s11934-021-01050-0

**Published:** 2021-04-22

**Authors:** Michele Marchioni, Juan Gomez Rivas, Anamaria Autran, Moises Socarras, Simone Albisinni, Matteo Ferro, Luigi Schips, Roberto Mario Scarpa, Rocco Papalia, Francesco Esperto

**Affiliations:** 1 Unit of Urology, Department of Medical, Oral and Biotechnological Sciences, SS. Annunziata Hospital, “G. d’Annunzio University”, Chieti, Italy; 2 Department of Medical, Oral and Biotechnological Sciences, University “G. D’Annunzio” Chieti-Pescara, Via dei Vestini, Campus universitario, 66100 Chieti, Italy; 3 Hospital Clinico San Carlos, Madrid, Spain; 4 Department of Urology, Fundacion Jimemez Diaz, Madrid, Spain; 5 Instituto de Cirugia Urologica Avanzada (ICUA), Madrid, Spain; 6 Urology Department, Université Libre de Bruxelles, Erasme Hospital, Brussels, Belgium; 7 Department of Urology, European Institute of Oncology (IEO), IRCCS, Milan, Italy; 8 Department of Urology, Campus Bio-Medico University, Rome, Italy

**Keywords:** Renal cell carcinoma, miRNA, Biomarkers, Liquid biopsy

## Abstract

**Purpose of Review:**

We aim to summarize the current state of art about the possible use of biomarkers for predicting renal cell carcinoma (RCC) recurrence after curative treatment. In addition, we aim to provide a snapshot about the clinical implication of biomarkers use for follow-up planification.

**Recent Findings:**

A wide variety of biomarkers have been proposed. RCC biomarkers have been individuated in tumoral tissue, blood, and urine. A variety of molecules, including proteins, DNA, and RNA, warrant a good accuracy for RCC recurrence and progression prediction. Their use in prediction models might warrant a better patients’ risk stratification.

**Summary:**

Future prognostic models will probably include a combination of classical features (tumor grade, stage, etc.) and novel biomarkers. Such models might allow a more accurate treatment and follow-up planification.

## Introduction

Renal cell carcinoma (RCC) represents about the 5% of all malignancies in men and about the 3% in women [1]. The most common subtype is clear cell RCC (ccRCC) that accounts for more than 60% of all RCC [2]. In addition, more than 65% of all RCC are diagnosed as localized, and 5-year survival rates are over 90% in those with organ-confined tumors [1]. Diagnoses of RCC often occur in the sixth decade of life, during exams (i.e., abdomen imaging) performed for other reasons or within screening programs, and at an early stage (< 4 cm of diameter). Such small renal masses are often indolent, and in the last few years, awareness about the potential harm derived from overdiagnosis and overtreatment of patients diagnosed with kidney cancer is rising [3].

Potential harms from overdiagnosis and overtreatment could derive from psychosocial stress, financial toxicity, unknown survival benefit, and treatment-related injuries (i.e., loss of kidney function and surgical complications) [3]. In addition, these patients have to be followed for at least 5 years after primary treatment, with different schedules based on their own oncological risk [4].

Indeed, recurrences after curative treatment, either partial or radical nephrectomy, are possible. A recent analysis by Dabestani et al. of the RECUR database has shown 5-year cumulative recurrence rates ranging from 7.2 to 61.6% in patients with ccRCC [5]. The median time to recurrence ranged from 12.5 to 43.7 months according to different risk categories [5]. These evidences corroborate the need for a long term follow-up as currently suggested by guidelines [4]. Furthermore, follow-up strategies should be adapted based on the patients’ own risk.

Unfortunately, follow-up schedules and strategies suggested by different scientific societies differ substantially. Several uncertainties have been shown in terms of duration, protocol, and impact of follow-up protocols on individual patients and society [6]. Further uncertainty is derived from the absence of a clear guideline about the model to be used for patients’ stratification [6]. Several nomograms or prognostic categories have been suggested [7]. All of them used a combination of patient’s and tumor’s characteristics, with particular regard to the tumor stage, size, and grade [7]. Externally validated tools, showing a fair accuracy (C-index over 75%), are the Kattan nomogram, the University of California-Los Angeles Integrated Staging System (UISS) score, and the Leibovich risk model [6].

In the last few years alongside to clinicopathological features, several biomarkers have been proposed to predict the risk of RCC recurrence [8–10]. The combination of clinical, pathological, and biological markers showed to have good accuracy predicting survival outcomes in RCC [11]. We aim to summarize the current state of art about the possible use of biomarkers for predicting RCC recurrence after curative treatment. In addition, we aim to provide a snapshot about the clinical implication of biomarkers use for follow-up planification.

## Renal Cell Carcinoma Biomarkers

In the last few years, researchers have demonstrated the potential role of several biomarkers for RCC. Existing biomarkers could be classified according to the origin site as tissue-, blood-, or urine-based biomarkers [11]. In the following paragraphs, we resume the possible role of these biomarkers, including liquid biopsy, on the prediction and early diagnoses of recurrences and/or progression to metastatic status in RCC patients. We focused on studies published during the last 2 years.

### Tissue-Based Biomarkers

Histological subtype represents the most important feature to predict recurrence. In a recent analysis based on RECUR database, Abu-Ghanem et al. showed that 5-year recurrence free survival rates significantly differ between ccRCC, papillary RCC (pRCC), and chromophobe RCC (chRCC) [12]. More specifically, 78% of ccRCC patients were disease free at 5 years versus 86% of pRCC and 91% of chRCC (*p* = 0.001). The association between histological subtype and recurrence rates remained statistically significant even in multivariable models taking into account the tumor stage, size, nuclear grade, vascular invasion, and surgical margin status [12]. It is of note that not only the recurrence rates were significantly different among different histological subtypes, but also the pattern of recurrence was significantly different. In particular, ccRCC recurred more frequently at lung. Conversely, pRCC showed a tropism for lympatic recurrence. Furthermore, chRCC had low recurrence rate, but liver and bone metastases were more frequent. Moreover, those with curable disease at recurrence harbored more frequently chRCC [12].

A correlation between RCC recurrence and/or progression with a wide series of tissue biomarkers has been also reported. In a recent analysis, Solano-Itturi and colleagues investigated the effect of fibroblast activation protein-α (FAP) expression on development of early metastases and cancer specific survival. Authors showed that FAP is expressed on fibroblast surface in tissue samples of ccRCC, pRCC, and chRCC but not in renal oncocytoma. Authors also showed that high expressions of FAP are associated with development of early metastases and worse cancer-specific survival. Interesting FAP soluble isoform levels were lower in samples from renal tumors than in controls [13]. Similarly, Wu et al. showed that high expression of TYROBP, a gene closely related to immune cell infiltration and co-expressed with programmed cell death protein-1 (PD-1) and cytotoxic T lymphocyte–associated antigen-4 (CTLA-4), was associated with low survival rates in ccRCC [14].

The role of nuclear renin expression was investigated by de Almeida e Paula and colleagues in 498 patients with nonmetastatic ccRCC who were treated with radical or partial nephrectomy. Authors showed that the qualitatively and quantitative negative renin expression was an unfavorable prognostic factor for disease-free survival. Authors hypothesized that renin expression decline could be due to direct structural and functional dysregulations. In particular, dysregulation of granular cells could be associated to alterations in mammalian target of rapamycin (mTOR) and Von Hippel-Lindau (VHL) signaling pathways [15]. These two pathways are well known to be involved in RCC pathogenesis, and expressions of proteins involved in these pathways were previously associated with worse survival outcomes in patients with RCC [11]. In a recent study, Wierzbicki et al. showed that high mRNA and protein expression of hypoxia-inducible factor 2α and vascular endothelial growth factor A were associated with shorter progression-free survival. Moreover, the expression of these two biomarkers was associated with sunitinib resistance [16]. Those findings strengthen the importance of these pathways in tumor progression and corroborate their renowned role in RCC prognosis. However, the mechanisms regulating cell proliferation and tumor progression seem to be more complex. Another recent analysis focused on the role of hepatocyte factor-4α [17]. Authors found that the downregulation of this protein promotes cell migration and invasion by transcriptional regulation of E-cadherin in RCC [17].

Ubiquitin-specific protease 2 expression was investigated by Meng et al. in ccRCC. Authors showed that aberrant expression of ubiquitin-specific protease 2 mRNA acted as independent prognosticator for ccRCC (AUC: 0.89, *p* < 0.001) [18]. Moreover, authors showed an association of its expression and disease-free survival. Indeed low levels of this biomarker were associated with lower rates of disease-free survival (HR: 0.67, *p* = 0.037) within models adjusting for age, gender, T-stage, N-stage, M-stage, and grade [18].

Furthermore, the importance of immune response to RCC is well known. Immune check point inhibitors have shown high efficacy in advanced renal cell carcinoma [19, 20]. Several reports showed that the expression of programmed death 1 (PD-1) and its ligand PD-L1 are associated with survival outcomes. In particular, PD-1 and PD-L1 expressions have been associated with adverse ccRCC features and poor outcomes in patients with advanced RCC [21••]. Other biomarkers associated with immune specific response to tumor have been proposed. Xiong et al. studied the effect of hypoxia-inducible factor 2α on CD8^+^ T cells [22]. According to the authors, the hypoxia-inducible factor 2α might improve the expression of chemo-attractive factors for mast cells. These mast cells could impair anti-tumor immunity secreting IL-10 and TGF-β [22]. In another recent study, Strizova et al. showed that peritumoral tissue of ccRCC patients is a reservoir of NK and T cells [23]. Conversely, non-ccRCC tumors had a significantly decrease in tumor infiltration by NK cells [23].

Among the others, also microRNAs (miRNAs) have been investigated as possible biomarkers of recurrence and progression in patients affected by RCC [24]. miRNAs are small non-coding RNAs that mediate gene expression trough mRNA cleavage and translation repression [24]. The main function of miRNA is as tumor suppressors [24]. Saleeb et al. showed that miR-200b- and miR-200c-positive patients have longer disease-free survival. Authors also showed that disease-free survival rates were better predicted when 2 or more miRNA are used in a combination. miR-200 family targets are associated with pathways related to cancer invasion and metastasis [24]. Another potential cluster has been individuated by Yuan and colleagues. In particular, the product of the miR-183/182/96 gene cluster was associated with worse overall survival. Authors concluded that this pathway should be investigated in future studies to shed a light on its possible role as biomarkers of tumor progression [25]. Similar to miRNA, circular RNA (circRNA) is an endogenous RNA which was reported to act as a possible regulator in several type of cancers. In particular, cRAPGEF5, a circRNA derived from exons 2–6 of the RAPGEF5 gene, have been observed to be downregulated in patients with shorter recurrence-free survival time [26]. In particular, cRAPGEF5 suppress RCC proliferation and migration [26].

### Liquid Biopsy (Blood-Based and Urinary Biomarkers)

Liquid biopsy is an emerging minimally invasive tool to discern possible cancer markers in biological liquids such as blood or urine. We now refer to the complex of proteins and other potential biomarkers, such as circulating tumor cells, circulating tumor DNA or RNA, extracellular vesicles, and metabolites, as “circulome” [8••]. Liquid biopsies have a series of advantages over tissue biopsies. First, the minimally invasiveness enables to perform an easier, safer, and earlier evaluation stratification and prediction of individual patient risk [27]. Second, liquid biopsies can be easily repeated allowing a continuous monitoring of residual disease and prognosis [27]. Third, monitoring allows to a prompt switch from a therapeutic approach to another [27]. Fourth, there is no need for hospitalization, thanks to the faster sampling and analysis [27]. All together, these aspects allow to a significant cost reduction [27]. All these peculiar characteristics are the reasons why liquid biopsy seems to have a great appealing. To date, only a small number of non-invasive blood tests, based on circulating DNA, are used. However, recently, several novel and promising circulating biomarkers have been reported to allowing the identification of disease recurrence in ccRCC, as highlighted in a recent literature review [8••]. Here, we will report only a selection of the most promising circulating biomarkers reported in the last few years in literature.

The introduction of next-generation sequencing allowed to the diffusion of circulating tumor DNA-based methods. Circulating tumor DNA offer all the advantages of liquid biopsy over tissue biopsies. In particular, circulating DNA have demonstrated to be a potential biomarker for recurrence. A recent literature review by Bergerot et al. reported results from several studies about the use of circulating tumor DNA [27]. In particular, circulating tumor DNA showed a potential as surveillance biomarker in the localized RCC setting and as a good forecaster for early diagnosis of metastases. In addition, authors reported results from studies showing changes in the mutational profile in circulating tumor DNA, evolving after treatment progression. But even more interesting was to notice that the tumor mutational burden is a predictor of response to immunotherapy [27]. Unfortunately, the main limitation of reported studies stands in the small sample size that reduces the generalizability of their findings.

Another interesting biomarker is represented by circulating tumor cells (CTC). These cells are cancer cells emitted from the tumors in the bloodstream recognized as mediator cancer metastases [28]. Recent studies showed the presence of CTC in ccRCC and non-ccRCC, with a slightly higher prevalence in ccRCC [29]. Moreover, the average size of primary tumor diameter was higher in patients with positive CTC [29]. Larger tumors were also more frequently metastatic or showed lymph node invasion [29]. It is of note that different CTC subtypes have been identified based on molecular expression patterns. A recent study pointed out that different CTC subtypes were able to predict inferior vena cava invasion [30]. Similarly, another study showed that CTC were associated with the metastatic status in patients with RCC.

However, authors also showed that differences in terms of CTC subtypes and its variation trend [31]. Unfortunately, to date, even if the use of CTC as possible biomarker of recurrence or tumor progression in RCC patients is of great appeal, available evidences suggest that its use in clinical practice is still far to come [32].

Among blood-based biomarkers, circulating RNAs have been also explored. In particular, non-coding RNAs (ncRNAs), which included also microRNAs (miRNAs) and long non-coding RNAs (lncRNAs), have been studied as possible biomarkers in several tumors [33]. Moreover, miRNAs could be detected also within exosomes that are vesicular cargos involved in the crosstalk between cells. In particular, exosomal miRNAs might have a role in numerous pathways involved in tumor progression and chemoresistance [34]. However, the role of these ncRNAs as potential biomarkers in RCC is still under evaluation, and in absence of validation in well-designed studies, as well as in absence of standardization of sample processing and normalization, circulating ncRNAs only represent a field for future researches [33].

Urinary miRNA have been investigated in several studies showing a promising role as biomarker candidates for RCC [35]. In particular, miRNA have shown the most promising characteristics in RCC diagnosis. Conversely only few studies explored the role of urine miRNA for RCC monitoring and none for RCC prognosis prediction [35]. However, also for urinary biomarkers, a validation and standardization are still far from clinical practice.

### Clinical Implication From the Use of Biomarkers in Renal Cell Carcinoma

Potentially, reliable biomarkers could allow to a better stratification of patients affected by RCC and treated with curative intent. In particular, a more specific knowledge of recurrence pattern could allow to tailor the imaging modalities used for follow-up based on the primary tumor characteristics. For instance, patients diagnosed with pRCC, who showed an apparent tropism for lymphatic recurrences, might be those who benefit the most from abdominal cross-sectional imaging after surgery [12]. Moreover, such lymphatic trophism raises questions about the possibility to use routine lymph node dissection in patients with pRCC [12]. Previous studies showed no clear survival advantage from lymph node dissection in patients with high-risk RCC [36]; however, no specific analysis was performed according to different histological subgroups.

The use of integrated models, including proposed biomarkers, might improve the accuracy of such models. For instance, renin expression, as well as other known covariates, namely symptoms at diagnosis, tumor size, pathological stage, and AJCC clinical stage, remained independent predictors of disease-free survival in multivariable Cox’s regression models [15]. Unfortunately, authors did not report any accuracy metric of their model including or not renin expression, and no external validation of their models has been performed [15]. Still, the importance of renin as covariate remains, and future studies should investigate its possible role as prognosticator, alone or in combination with other biomarkers. In the current review of the literature, we reported the high accuracy of ubiquitin-specific protease 2 expression predicting disease-free survival [18]. Indeed, a fair prediction ability was shown for ubiquitin-specific protease 2 expression [18]. Moreover, a recent study relied on machine learning methods to identify molecular biomarkers associated with aggressive cT1 ccRCC. Authors showed that when three or six parameters were included in the model, the use of deep neural network models exerted a better performance compared to logistic regression models as shown by the area under the curve. Indeed, area under the curve ranged between 65.1 and 76.0% when logistic regression models were used versus 73.6 and 79.6% when deep neural network models were employed [37]. Similarly, Grimm et al. relied on an algorithm for risk stratification of metastases in patients with ccRCC [38]. The algorithm combined the total number of specific aberration genetic score and T-category. The combination of both showed an increase of the prognostic accuracy to 87%. The model was able to classify the patients in two risk groups with different recurrence free survival, cancer specific survival, and overall survival [38]. The model was also better than the Leibovich risk group classification system (C-index 0.848 vs. 0.742) [38].

These results suggest that a further improvement in development of integrated classification risk models could derive both from the identification of new biomarkers and the use of advanced analytical methods. Bioinformatic is fundamental in gene expression analysis and to analyze microarrays data. Bioinformatics is a growing field of research commonly used to identify candidate genes useful to the compression of genetic disease bases [39]. Zhou et al. were able to evaluate and validate the value of CEP55 in ccRCC. In particular, CEP55 was associated with poor prognosis [39]. The use of bioinformatics techniques is essential in order to obtain results interpretable from datasets containing several hundreds of gene expression information.

The identification of novel biomarkers might open a new era in tailored medicine for RCC. In particular, new biomarkers might significantly improve early diagnosis and treatment planification [40]. In the last years, thanks to the introduction of novel therapeutic strategies, a significant improvement has been reported in terms of survival even in patients with advanced or metastatic RCC [41], a further improvement could derive by a better patients stratification and selection, thanks to these novel tools. However, sample acquisition, storage, and analysis could be a main limitation for the routinely use of such biomarkers [40]. As a consequence, identification and validation of RCC biomarkers represent only the first phase of a complex process. A significant source of variability in the pre-analytical and analytical phase could affect the accuracy and reproducibility of the obtained results, limiting their implementation and development [40]. So a strict standardization of all the procedure and of the overall assay should be warranted in order to achieve an accurate and reliable quantification of these biomarkers [40].

A further help to physicians might also come from a more accurate imaging. Indeed, besides tumor size, also enhancement characteristics, tumor margin, and distance to renal sinus at computed tomography imaging have been associated with features predicting biological aggressiveness of RCC [42]. These preoperative features, as well as those already known to be associated with survival, such as positive surgical margins (PSM), might be integrated in future tools including also molecular biomarkers [43]. Finally, more accurate predictive tools could be fundamental for stratifying patients eligible to active surveillance for small renal masses. During last years, a growing interest emerged for such therapeutic strategy, and active surveillance showed survival outcomes that are comparable to active treatment [44, 45]. Unfortunately, to the best of our knowledge, only a scoring system applies specifically to active surveillance patient selection [46]. On the other hand, a deeper knowledge of molecular mechanism involved with systemic treatment resistance might be helpful for selecting patients at highest risk, such as those with locally advanced tumors [47], who might benefit of an adjuvant or neo-adjuvant treatment [48].

## Conclusion

Several biomarkers have been proposed in the last few years for early prediction of RCC progression and monitoring. These biomarkers are various in nature and have been identified in tumoral tissue, plasma, serum, or urine. Prognostic models including a combination of these biomarkers alongside to classical well-known features might be more accurate, compared to those which are currently available. So patients’ stratification could be improved, allowing a more tailored treatment choice, based on biomolecular tumor and patient characteristics.
